# The Spatio-Temporal Distribution of Cell Wall-Associated Glycoproteins During Wood Formation in *Populus*

**DOI:** 10.3389/fpls.2020.611607

**Published:** 2020-12-15

**Authors:** Tayebeh Abedi, Romain Castilleux, Pieter Nibbering, Totte Niittylä

**Affiliations:** Department of Forest Genetics and Plant Physiology, Umeå Plant Science Centre, Swedish University of Agricultural Sciences, Umeå, Sweden

**Keywords:** hydroxyproline-rich glycoproteins, arabinogalactan-proteins, β-D-glucosyl Yariv, extensins, *Populus*, wood formation

## Abstract

Plant cell wall associated hydroxyproline-rich glycoproteins (HRGPs) are involved in several aspects of plant growth and development, including wood formation in trees. HRGPs such as arabinogalactan-proteins (AGPs), extensins (EXTs), and proline rich proteins (PRPs) are important for the development and architecture of plant cell walls. Analysis of publicly available gene expression data revealed that many *HRGP* encoding genes show tight spatio-temporal expression patterns in the developing wood of *Populus* that are indicative of specific functions during wood formation. Similar results were obtained for the expression of glycosyl transferases putatively involved in HRGP glycosylation. *In situ* immunolabelling of transverse wood sections using AGP and EXT antibodies revealed the cell type specificity of different epitopes. In mature wood AGP epitopes were located in xylem ray cell walls, whereas EXT epitopes were specifically observed between neighboring xylem vessels, and on the ray cell side of the vessel walls, likely in association with pits. Molecular mass and glycan analysis of AGPs and EXTs in phloem/cambium, developing xylem, and mature xylem revealed clear differences in glycan structures and size between the tissues. Separation of AGPs by agarose gel electrophoresis and staining with β-D-glucosyl Yariv confirmed the presence of different AGP populations in phloem/cambium and xylem. These results reveal the diverse changes in HRGP-related processes that occur during wood formation at the gene expression and HRGP glycan biosynthesis levels, and relate HRGPs and glycosylation processes to the developmental processes of wood formation.

## Introduction

Xylem formation in trees is initiated in the vascular cambium and proceeds through cell expansion, secondary cell wall deposition, maturation, and programmed cell death, culminating in heartwood formation (Plomion et al., 2001). The xylem of angiosperm trees, commonly known as wood, contains fibers that provide mechanical support, water conducting vessels, and ray cells involved in radial transport, storage, and heartwood formation (Déjardin et al., 2010). During cell expansion, the xylem cell walls consist of three main polymers: cellulose, hemicelluloses, and pectin. These polysaccharides together with cell wall associated proteins form a complex extendable matrix called the primary cell wall. The properties of the primary cell wall control cell expansion and the direction of growth, influencing xylem fiber length and vessel dimensions (Plomion et al., 2001; Rose and Lee, 2010). Once the cells have reached their final size, a secondary cell wall composed primarily of cellulose, hemicelluloses, and lignin is synthesized on top of the primary cell wall on the inner side of the fibers and vessels (Höfte and Voxeur, 2017). In addition to the cell wall polymers, several classes of glycoproteins with structural and signaling functions are involved in cell wall formation (Rose and Lee, 2010). In particular the cell wall associated glycoproteins known as hydroxyproline-rich glycoproteins (HRGPs) are thought to influence the synthesis and properties of both primary and secondary cell walls. HRGPs are also found in wood, but their role during the secondary growth of trees is largely unknown (Plomion et al., 2001).

HRGPs are the main class of cell surface glycoproteins in plants that have been linked to cell wall assembly and cell architecture. These complex macromolecules exhibit high structural and functional diversity, and play central roles in plant growth, development, and adaptation to changing environmental conditions (Hijazi et al., 2014; Jiao et al., 2018). It is thought that they perform these roles by modifying the physical and chemical properties of the cell wall in response to developmental and environmental signals (Seifert and Roberts, 2007; Hijazi et al., 2014). HRGPs are highly diverse but can be divided into three main subfamilies based on their proline hydroxylation patterns and glycosylation: the highly glycosylated arabinogalactan-proteins (AGPs), the moderately glycosylated extensins (EXTs), and proline-rich proteins (PRPs) that may be non-, weakly-, or highly glycosylated (Hijazi et al., 2014). Because of their diverse and repetitive protein motifs, bioinformatics approaches have been used to characterize and study this complex family. A total of 271 HRGPs have been identified in the model tree poplar (*Populus trichocarpa*), including 162 AGPs, 60 EXTs, and 49 PRPs (Showalter et al., 2016).

AGPs and EXTs are abundant during primary cell wall biosynthesis (Tan et al., 2018). Their biological roles may depend on the characteristics of both their protein core and the attached glycans (Cannon et al., 2008). AGPs are found on the surfaces of plasma membranes, where they are attached via a glycosylphosphatidylinositol (GPI) membrane anchor, or in the membrane-cell wall interspaces or in the cell wall matrix. They are found in many tissues but are especially abundant in xylem (Nothnagel, 1997; Showalter, 2001). At the organ level, AGPs are found everywhere including in leaves, stems, roots, floral parts and seeds. The AGP protein backbone undergoes multiple phases of post-translational modification in the ER and Golgi apparatus, typically involving hydroxylation of proline residues and often the covalent addition of a GPI anchor at the C terminus (Borner et al., 2003; Ellis et al., 2010). The GPI anchor is thought to be important for AGPs involved in signaling pathways (Schultz et al., 1998). The glycan moieties of AGPs, which typically account for 90–98% of their total molecular mass, are *O*-linked to hydroxyproline residues (and possibly also serine and threonine residues) in the protein core by various glycosyltransferases (GTs) (Ellis et al., 2010). Analyses of AGP glycans isolated after alkaline hydrolysis have shown that the AG polysaccharide chains vary in size from 30 to 150 sugar residues (Tsumuraya et al., 1984; Qi et al., 1991). AG glycans are structurally complex, consisting of β-1,3-galactan main chains with β-1,6-galactan side chains of various lengths that are further decorated with arabinose and other sugars such as glucuronic acid, rhamnose, mannose, xylose, glucose, and fucose (Ellis et al., 2010; Tan et al., 2010; Kitazawa et al., 2013). Little is known about the sequences of these polysaccharide units or their structure-function relationships in AGP glycans. However, studies using the β-D-glucosyl Yariv reagent, which binds specifically to the β-1,3-galactan moiety of AGPs (Kitazawa et al., 2013), and various monoclonal antibodies that recognize different AGP glycan epitopes (Seifert and Roberts, 2007), have demonstrated that the glycans are essential for the function of AGPs. The diversity in the composition and the structure of AGPs may explain their multitude of biological functions, which includes wood formation in trees (Yang et al., 2005).

Extensins are the other main group within the HRGP family. These glycoproteins have a distinctive motif consisting of several consecutive *O*-glycosylated serine-(hydroxyprolines). The hydroxylation of proline residues by prolyl-4-hydroxylases and the addition of a galactose onto the adjacent serine residue by serine-galactosyltransferase 1 (SGT1) both occur in the endoplasmic reticulum (Fragkostefanakis et al., 2014; Saito et al., 2014; Velasquez et al., 2015; Marzol et al., 2018). Then, in the Golgi apparatus, several arabinoses are successively transferred to the hydroxyproline residues (Velasquez et al., 2012; Moller et al., 2017). The glycans of EXTs are particularly important because they are thought to force the adoption of a conformation that permits intra- and/or intermolecular cross-linking of the protein component via tyrosine residues, resulting in the formation of isodityrosine, pulcherosine or di-isodityrosine linkages (Smith et al., 1986; Schnabelrauch et al., 1996; Held et al., 2004; Lamport et al., 2011; Chen et al., 2015; Velasquez et al., 2015). This cross-linking process is catalyzed by specific peroxidases (Brownleader et al., 1995; Schnabelrauch et al., 1996; Price et al., 2003; Dong et al., 2015; Marzol et al., 2018; Jacobowitz et al., 2019). EXTs are involved in many biological processes including cell expansion (Mravec et al., 2017) and cell wall assembly (Cannon et al., 2008; Lamport et al., 2011; Pereira et al., 2011; Chormova and Fry, 2016; Marzol et al., 2018). Extensin-associated epitopes were also found in the G-layer of poplar tension wood (Guedes et al., 2017; Decou et al., 2020) and genes encoding EXTs were upregulated in black pine stems in response to nematode inoculation (Hirao et al., 2012), however little is known about the function of EXTs in wood.

The PRPs are the third group of the HRGP family. The *O*-glycosylation rates of PRPs and their interactions with other cell wall components appear to be highly variable (Hijazi et al., 2014). Their amino acid sequences feature repeating units of 2–3 proline or hydroxyproline residues and are also rich in valine, lysine, and tyrosine (Showalter et al., 2016). They have been linked to various aspects of plant development, responses to hydric stress, plant defense, and cell wall strengthening (Bradley et al., 1992; Brisson et al., 1994; Battaglia et al., 2007; Chen et al., 2014). While their functions in the cell wall are largely unknown, a correlation between overexpression of PRP genes and changes in the microfibril angles in the secondary cell walls of poplar wood was recently reported (Li et al., 2019).

Here we investigate the expression of the *HRGP* family in *Populus* stems and their role in wood formation by performing an extensive bioinformatic and phylogenetic analysis combined with an analysis of genes encoding enzymes associated with HRGP glycosylation. Further insights were provided by performing an immunochemistry analysis to determine the location of extensin and AGP epitopes in wood.

## Materials and Methods

### Bioinformatic Analysis

The HRGPs considered in this work and their nomenclature are derived from Showalter et al. (2016). Basic Local Alignment Search Tool (BLAST) analysis were performed using POPGENIE (Populus Genome Integrative Explorer^1^). Phylogenetic trees were constructed with full length protein sequences from the *Populus* Genome Integrative Explorer (see text footnote 1) database (Sjodin et al., 2009) and were created using the Molecular Evolutionary Genetics Analysis X (MEGA-X) software package (Kumar et al., 2018). The full-length protein sequences were first aligned with ClustalW using its standard settings (Thompson et al., 1994; Larkin et al., 2007). Phylogenetic analysis was then performed using the maximum likelihood method of MEGA-X in default mode with 1000 bootstrap replicates. The relative developing wood expression levels of the genes from *Populus tremula* in this study were obtained from the ASPWOOD database^2^ (Sundell et al., 2017). The ASPWOOD database provides interactive tools for analysis of gene expression profiles and co-expression networks obtained by sequencing of RNA from cryo-sectioned developing wood of *P. tremula*. Relative expression values from four biological replicates were averaged and heatmaps were generated accordingly using the R software.

### Plant Material and Growth Conditions

Hybrid aspen (*P. tremula* × *Populus tremuloides*) trees were micropropagated *in vitro* for 4 weeks and then transferred to a greenhouse for further growth in commercial soil with a fertilizer mixture (Hasselfors Garden Planteringsjord^3^) under an 18-h light/6-h dark photoperiod at a temperature of 22/15°C (light/dark) and 50–70% humidity. The trees were fertilized using 150 ml 1% Rika-S (N/P/K, 7:1:5; Weibulls Horto, SW Horto AB, Hammenhög, Sweden) once a week for the first 3 weeks of greenhouse growth.

### Immunolabelling on Wood Cross-Sections

Fifteen centimeters long stems of hybrid aspen *P. tremula* × *P. tremuloides* (T89) were collected from 10 cm above the soil after 3 months of growth in the greenhouse. Stems were frozen in liquid nitrogen and stored at −20°C, then rehydrated in distilled water at +4°C for a day or two. Thirty micrometers thick cross-sections were cut using a vibratome and placed on slides hydrated with 0.01 M phosphate-buffered saline (PBS). The sections were then fixed for at least 30 min in 4% (v/v) paraformaldehyde diluted in 0.01 M PBS buffer. After three washes with PBS buffer 0.01 M, they were incubated overnight at +4°C in a wet chamber with a primary monoclonal antibody (mAb) from PlantProbes^4^ or CarboSource Services^5^, diluted at 1:10 in a solution of 5% (w/v) milk protein in 0.01 M PBS (see list of the anti-AGPs and anti-extensin mAbs used in Supplementary Table 1). The sections were then washed three times with PBS 0.01M, after which they were incubated for 2 h at room temperature in a wet chamber with the secondary antibody anti-rat IgG DyLight 550 (Agrisera, AS12 1973) diluted at 1:50 in a solution of 5% (w/v) milk protein in 0.01 M PBS. After three final washes with PBS 0.01M, the slides were covered and the sections were observed with a Zeiss LSM 780 inverted confocal microscope (λ_excitation_: 514 nm; λ_emission_: 535–650 nm) using the same photomultiplier tube value and exposure on each occasion. Each immunolabelling experiment was repeated at least three times using sections from at least three different trees. A “green fire blue” filter was applied to all fluorescence images using the Fiji software^6^ (Schindelin et al., 2012).

### Western Blot Analysis of AGPs and EXTs

Different stem parts involved in wood formation, namely the phloem/cambium, developing xylem, and mature xylem were collected separately by scraping stems from five individual trees. Materials were flash-frozen in liquid nitrogen, lyophilized, and ball milled. Water-soluble AGPs and EXTs were then extracted from pooled samples of each stem part using water at 50°C for 30 min. Western blot analysis was done as described previously (Fragkostefanakis et al., 2012). SDS-PAGE was performed to separate proteins according to Sambrook et al. (1989), after which the gels were transferred to a polyvinylidene difluoride (PVDF) membrane at 12 V at 4°C overnight. The membrane was then blocked in TBST buffer (10 mM Tris–HCl, 150 mM NaCl, 0.1% Tween-20, pH 7.6) containing 5% (w/v) milk powder for 1 h, followed by labeling with primary anti-AGPs mAbs (JIM8, JIM13, JIM14, JIM16, LM2, LM14, and MAC207) and primary anti-EXTs mAbs (JIM12, JIM19, JIM20, and LM1), from PlantProbes (see text footnote 4) or CarboSource Services (see text footnote 5), diluted 1:5000 in TBST buffer containing 2.5% (w/v) milk powder for 1 h. The labeled membranes were washed three times for 5 min each with TBST and then incubated with a 1:10000 dilution of anti-rat antibodies coupled to HRP for 1 h (Agrisera, AS10 1187). After a similar washing step, the blots were developed with the ECL prime western blotting detection reagent (Amersham Biosciences) according to the manufacturer’s protocols.

### Detection of AGP Subpopulations Using Agarose Gel

Detection of AGPs on agarose gel was performed according to Castilleux et al. (2020). Water-extracted AGPs from 15 mg pooled samples were loaded onto a 1% (w/v) agarose gel containing 90 mM Tris base pH 8.3 with HCl, 90 mM boric acid, and 2 mM Na_2_EDTA (H_2_O)_2_, and run at 100 V for 1 h. β-D-glucosyl Yariv was synthesized in house according to the protocol by Yariv et al. (1962). The gels were then stained with 10 μg β-D-glucosyl Yariv overnight, followed by destaining with 1% NaCl.

### AGP Quantification

Arabinogalactan-proteins quantification was done on five biological replicates according to Lamport (2013). Samples (2 mg) were mixed with 500 μl 2% CaCl_2_ and 200 μl β-D-glucosyl Yariv dissolved in 2% CaCl_2_ (1 mg/ml), then stirred for 2 h at room temperature. Gum arabic (10 or 20 μg) was used as an AGP standard. The β-D-glucosyl Yariv precipitate was collected by centrifugation at 15,000 × *g* for 10 min and washed twice with 2% CaCl_2_. The pellet was then dissolved in 20 mM NaOH, after which the dissolved AGPs were quantified by measuring their absorbance at OD457.

## Results and Discussion

### Phylogeny of *Populus* HRGPs and Their Expression During Wood Development

**FIGURE 1:**
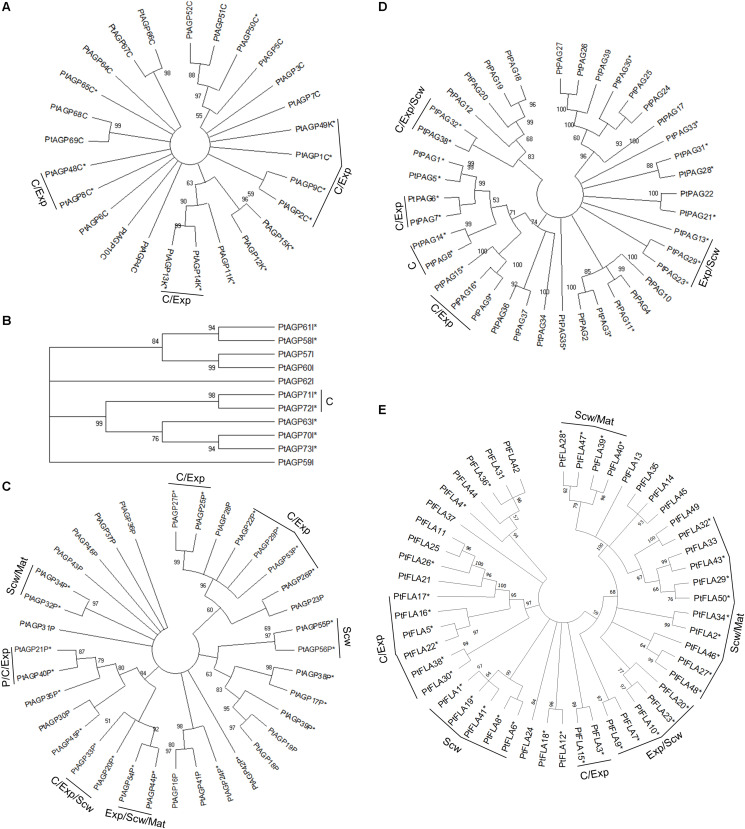
Phylogenetic tree of AGPs from *Populus trichocarpa.* The amino acid sequences of classical AGPs and lysine rich AGPs **(A)**, other chimeric AGPs **(B)**, AGP peptide **(C)**, plastocyanin AGPs **(D)**, and fasciclin-like AGPs **(E)** identified in the study of Showalter et al. (2016) were aligned by ClustalW. Phylogenic trees were constructed using the maximum likelihood method of MEGA-X in default mode with bootstrap test of 1000 replicates. The numbers beside the branches correspond to % bootstrap values. The asterisk (*) next to the gene name indicates expression in the wood. P, phloem; C, cambium; Exp, xylem expansion zone; Scw, xylem secondary cell wall formation zone; Mat, xylem maturation zone.

**FIGURE 2:**
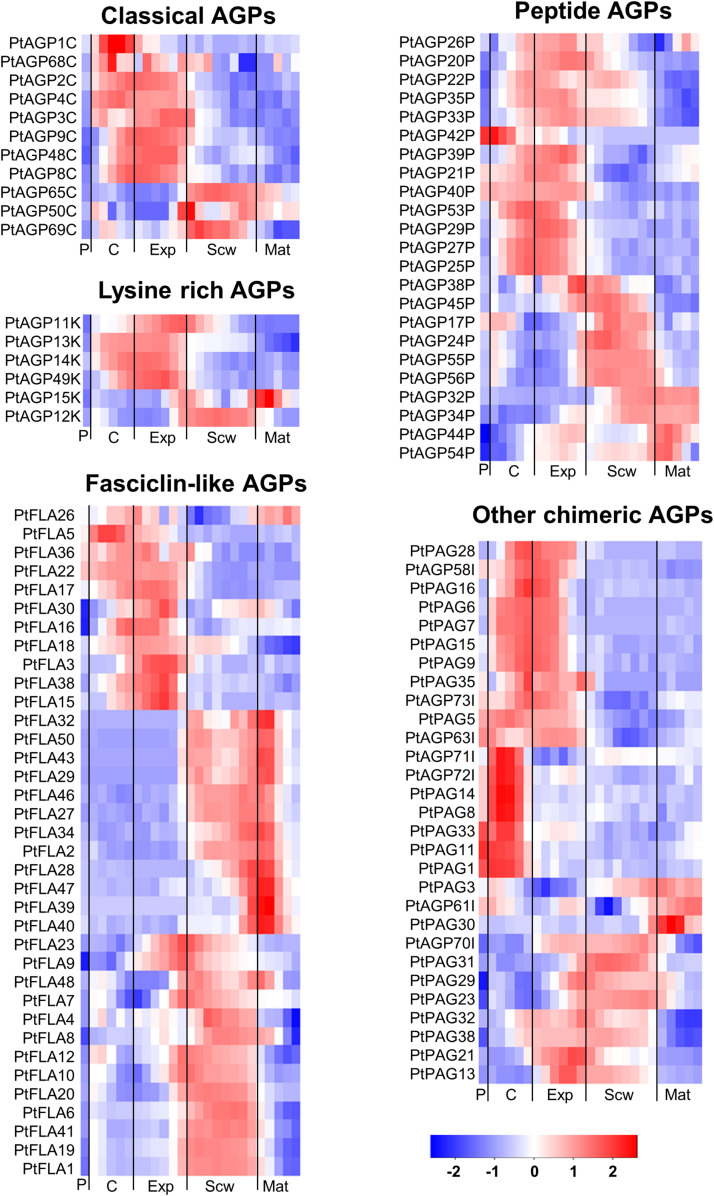
Heatmap of AGP expression in the wood of *Populus tremula.* Heatmap depicting the relative expression of AGPs in the phloem (P), cambium (C), xylem expansion zone (Exp), xylem secondary cell wall formation zone (Scw), and xylem maturation zone (Mat). Expression values are scaled per gene so that expression values above the gene average are shown in red and below average in blue. *n* = 4 biological replicates.

Arabinogalactan-proteins can be subdivided into different classes based on their amino acid sequence and domain structure. The currently recognized classes are classical AGPs, AG peptides, fasciclin-like AGPs (FLAs), plastocyanin AGPs (PAGs), lysine-rich AGPs, and other chimeric AGPs (Showalter et al., 2010). The classical AGPs (Figure 1A) showed little phylogenetic grouping due to their diverse amino acid sequences, domain structure and limited evolutionary expansion within *Populus.* The chimeric AGPs, lysine-rich AGPs, AGP peptides, plastocyanin and especially fasciclin-like AGPs (FLAs) formed more clear within class phylogenetic groups (Figure 1A–E), and interestingly some of these groups are associated with developmental stage specific gene expression during wood formation (Figures 1, 2).

Of the AGPs identified by Showalter et al. (2016), 11 out of 27 classical AGPs, 6 out of 6 Lysine-rich AGPs, 36 out of 50 FLAs, 23 out of 35 AG peptides, 22 out of 39 PAGs, and 7 out of 11 other chimeric AGPs are expressed during wood formation (see text footnote 2). We generated heatmaps showing the relative expression of each *HRGP* gene during wood formation (Figure 2). These heatmaps suggest that certain AGPs have specialized functions in defined zones during wood development (Figure 2). Most of the *AGP*s could be divided into groups based on the zone in which they exhibited the highest relative expression – the cambium/expansion, secondary cell wall formation, or cell wall maturation zones. Most *AGP*s were expressed quite weakly in the phloem with the exception of *PAG*s, which were strongly expressed in the phloem and cambium but weakly expressed in xylem. Similar expression profiles were observed for several *FLA*s that are phylogenetically close to each other, suggesting that their functions may be conserved (Figures 1E, 2). Studies on *AtFLA4* indicated that it is involved in cell expansion and cellulose biosynthesis in Arabidopsis under NaCl stress (Shi et al., 2003; Basu et al., 2016). Its close orthologs in *Populus*, *PtFLA16*, *30*, and *38* (Showalter et al., 2016), are strongly expressed in the expansion zone, suggesting that their function may be conserved between *Populus* and Arabidopsis (Figures 1E, 2). Similar results were obtained for several *FLA*s with high relative expression in the secondary cell wall formation and cell wall maturation zones (Figure 2). *AtFLA11* and *AtFLA12* are expressed strongly during secondary cell wall formation in Arabidopsis stems (Persson et al., 2005) and are close orthologs of the FLAs expressed during secondary cell wall formation in *Populus* (Figure 2; Showalter et al., 2016). *AtFLA11* and *AtFLA12* were proposed to be involved in cellulose deposition and to influence the mechanical properties of the secondary cell wall (MacMillan et al., 2010). Accordingly, RNAi-induced suppression of *AtFLA11/12* orthologs in *Populus davidiana* × *Populus bolleana* reduced cellulose and lignin levels and adversely affected the stem’s mechanical properties (Wang et al., 2015). The exact roles of FLAs in cell wall formation are poorly understood, but it has been proposed that at least some of them may function in cellulose biosynthesis. Among other things, FLAs could bind to cellulose in the cell wall matrix, affect cellular signaling leading to changes in cellulose biosynthesis, bind to cellulose synthase complexes (CSCs) and thereby either stabilize them or alter their function, or act as adaptors between CSCs and receptor kinases to modulate CSC activity (Seifert, 2018).

Most lysine-rich AGPs from *Populus* are expressed in the cambial and cell expansion zones of the wood (Figure 2). Lysine-rich AGPs in Arabidopsis have been associated with cell division and cell expansion (Ellis et al., 2010). For example, the megaspores of an Arabidopsis mutant defective in the lysine-rich AGP18 cannot grow and mitotically divide, showing that AGP18 is essential for female gametogenesis (Acosta-García and Vielle-Calzada, 2004). Similarly, silencing the expression of lysine-rich *AGP19* in Arabidopsis reduced the number of abaxial epidermal cells in rosette leaves, suggesting involvement in cell division (Yang et al., 2007). The mutant also had shorter hypocotyl cells, smaller rosette epidermal cells, and differently shaped mesophyll cells compared to the wild-type, all of which are indicative of cell expansion defects. The high relative expression of lysine-rich AGPs in the wood cambial and expansion zone suggests that they may function in cell division and/or cell expansion.

**FIGURE 3:**
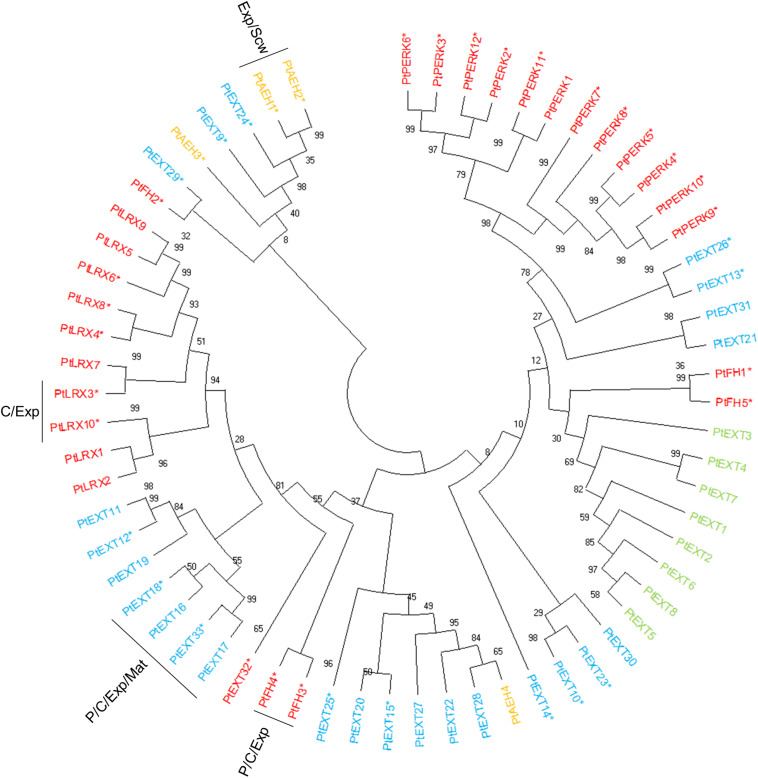
Phylogenetic tree of EXTs from *Populus trichocarpa.* The amino acid sequences of Classical EXTs (green), Short EXT (light blue), EXT/AGP hybrids (orange), and other chimeric EXTs (red) identified in the study of Showalter et al. (2016) were aligned by ClustalW. Phylogenic trees were constructed using maximum likelihood method of MEGA-X in default mode with bootstrap test of 1000 replicates. The numbers beside the branches correspond to % bootstrap values. The asterisk (*) next to the gene name indicates expression in the wood. P, phloem; C, cambium; Exp, xylem expansion zone; Scw, xylem secondary cell wall formation zone; Mat, xylem maturation zone.

**FIGURE 4:**
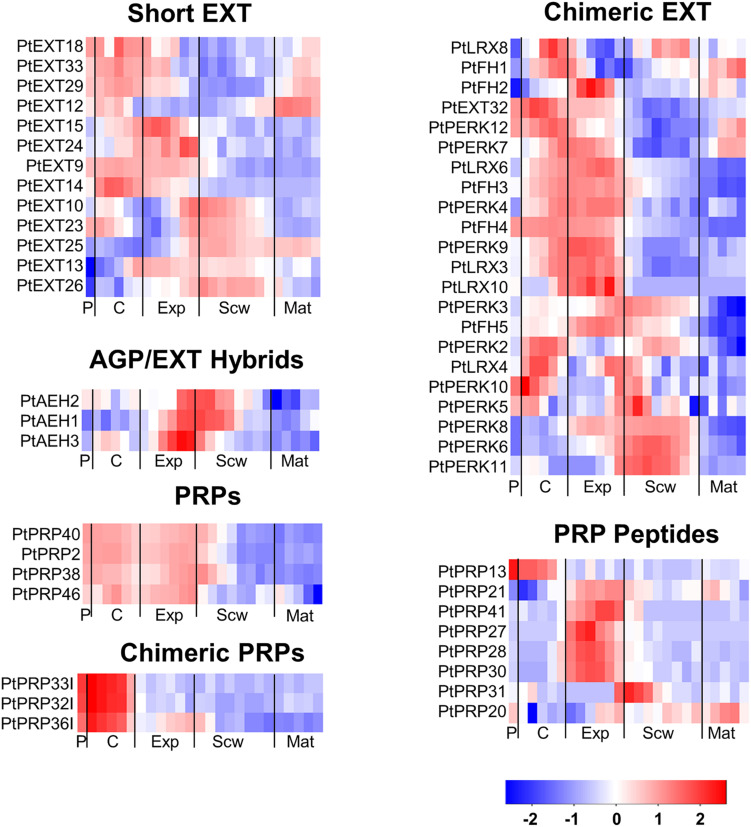
Heatmap of EXT and PRP expression in the wood of *Populus tremula.* Heatmap depicting the relative expression of EXTs and PRPs in the phloem (P), cambium (C), xylem expansion zone (Exp), xylem secondary cell wall formation zone (Scw), and xylem maturation zone (Mat). Expression values are scaled per gene so that expression values above the gene average are shown in red and below average in blue. *n* = 4 biological replicates.

Extensins can be subdivided into classical EXTs, short EXTs, chimeric EXTs and AGP/EXT hybrids (Showalter et al., 2016). Additionally, the chimeric LEUCINE-RICH REPEAT/EXTENSIN (LRX) and PROLINE-RICH EXTENSIN-LIKE RECEPTOR KINASE (PERK) proteins constitute two distinct groups in the phylogenetic tree (Figure 3). According to the ASPWOOD database, five of the ten annotated *Populus LRX*s were expressed in wood, mostly in the cambium and the expansion zone (Figure 4). LRXs are believed to be involved in cell elongation and to regulate the cell morphogenesis, although this function has mainly been studied in pollen and roots (Baumberger et al., 2001, 2003; Herger et al., 2019), which is consistent with the observation in this study that these proteins are mainly expressed in the cambium and expansion zone. The *PERK*s were found to be expressed throughout the wood (Figure 4). Interestingly, these proteins lack the Tyrosine-X-Tyrosine motif (where X is a variable amino acid) required for extensin cross-linking. In addition, they were proposed to be involved in regulating plant growth and development (Borassi et al., 2016). It is thus possible that PERKs may play a signaling role rather than a structural one during wood formation. The classical EXTs also clustered into a separate group in the phylogenetic tree (Figure 3), but surprisingly no classical *EXT* transcripts were detected in the developing wood of *P. tremula*. The short EXTs clustered into different groups, with some being closely related to the LRX, PERK, or classical EXT sequences. *PtEXT23*, *PtEXT10*, and the closely related short extensins *PtEXT13* and *PtEXT26* were mainly expressed in the secondary cell wall formation zone, but another phylogenetic group of short *EXT*s was expressed in the other wood developmental zones (Figures 3, 4). This implies that specific short EXTs are involved in secondary cell wall formation. As was also the case for the *AGP/EXT* hybrids, these short *EXT*s were expressed most strongly during the transition from cell expansion to secondary cell wall formation, and therefore may be involved in the cessation of expansive growth and initiation of cell wall thickening (Figure 4). Overall, short and chimeric *EXT*s exhibit high relative expression in well-defined wood development zones, like the AGPs, suggesting that they also have specific roles during wood development (Figure 4).

**FIGURE 5:**
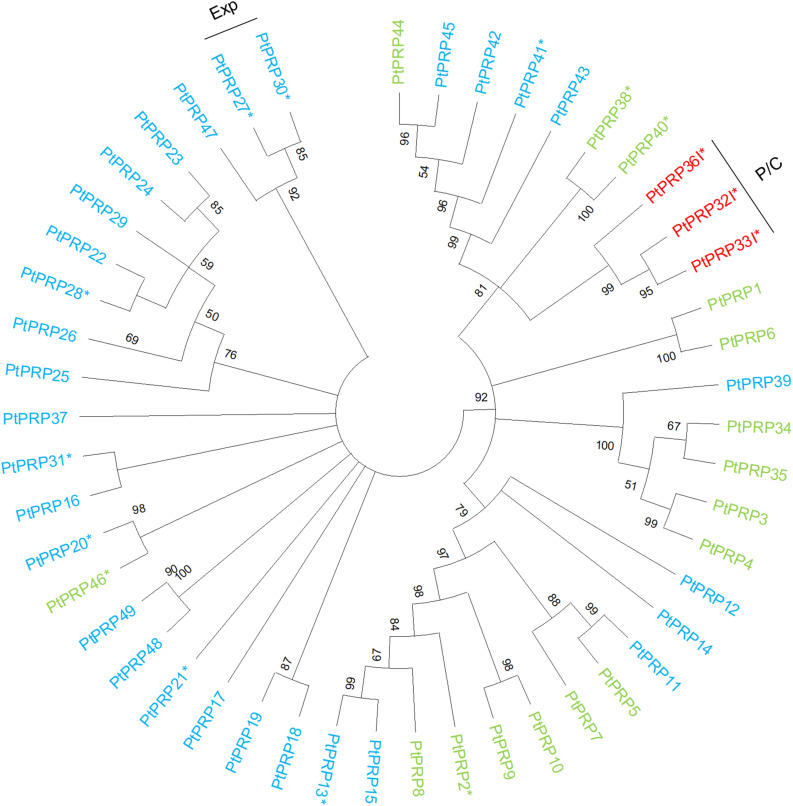
Phylogenetic tree of PRPs from *Populus trichocarpa.* The protein sequences of PRPs (green), PRP peptides (light blue), and chimeric PRPs (red) identified in the study of Showalter et al. (2016) were aligned via ClustalW. Phylogenic trees were constructed using maximum likelihood method of MEGA-X in default mode with bootstrap test of 1000 replicates. The numbers beside the branches correspond to % bootstrap values. The asterisk (*) next to the gene name indicates expression in the wood. P, phloem; C, cambium; Exp, xylem expansion zone.

The PRPs can be divided into PRPs, short PRPs and chimeric PRPs (Showalter et al., 2016). PRPs in general are more variable than other HRGPs, and some PRPs have protein sequence characteristics similar to those of EXTs or AGPs (Showalter et al., 2010). Like the *AGP*s and *EXT*s, *PRP*s are strongly expressed in specific wood developmental zones such as the phloem, cambium, and expansion zone (Figure 4). Interestingly, the phylogenetically close chimeric PRPs exhibit strong relative expression in the phloem and cambium (Figure 5). Conversely, most of the short *PRP*s are relatively highly expressed in the cell expansion zone. Based on these results, the PRPs probably have a role in the phloem, the cambium and the expansion zone of developing wood, while the expression data does not support a function in the secondary cell wall formation and wood maturation zones.

### Expression of Genes Encoding HRGP Glycosylating Enzymes in Developing Wood

**FIGURE 6:**
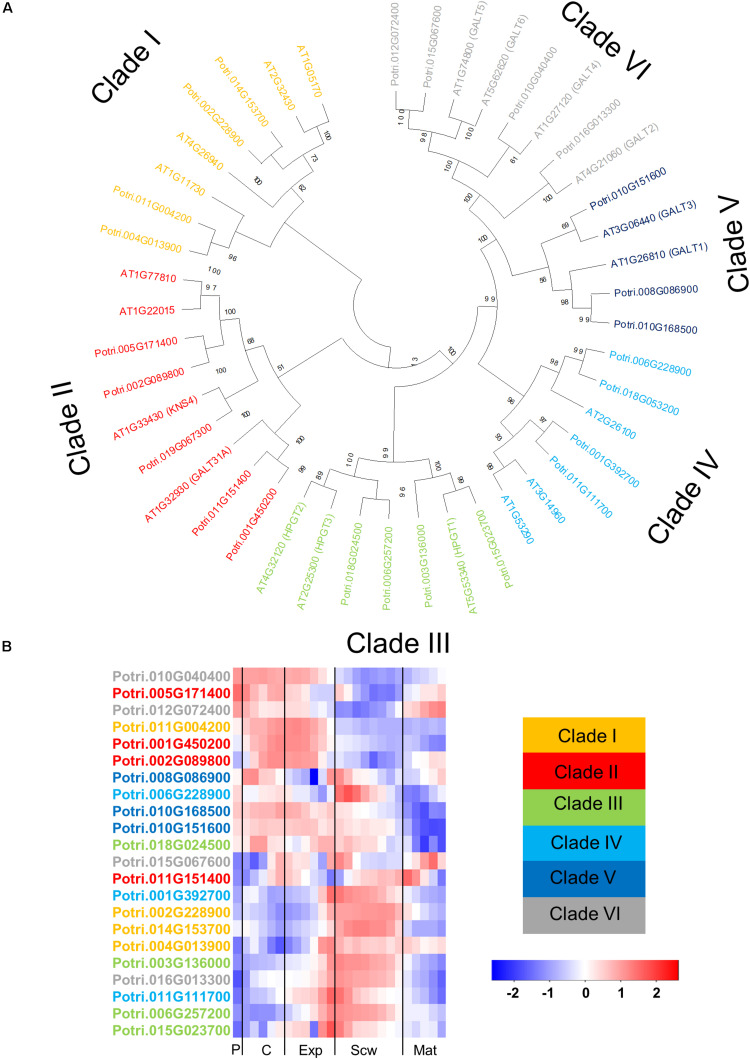
The phylogeny and expression of GT31 proteins in *Populus.* **(A)** Phylogenetic tree describing the phylogeny of the GT31 proteins from *Populus trichocarpa* and *Arabidopsis thaliana*. The proteins are divided into clades according to the study of Qu et al. (2008). Phylogenic trees were constructed using maximum likelihood method of MEGA-X in default mode with bootstrap test of 1000 replicates. The numbers beside the branches correspond to % bootstrap values. **(B)** Heatmap describing the relative expression of GT31 proteins from *Populus tremula* in the phloem (P), cambium (C), xylem expansion zone (Exp), xylem secondary cell wall formation zone (Scw), and xylem maturation zone (Mat). Expression values are scaled per gene so that expression values above the gene average are shown in red and below average in blue. *n* = 4 biological replicates.

**FIGURE 7:**
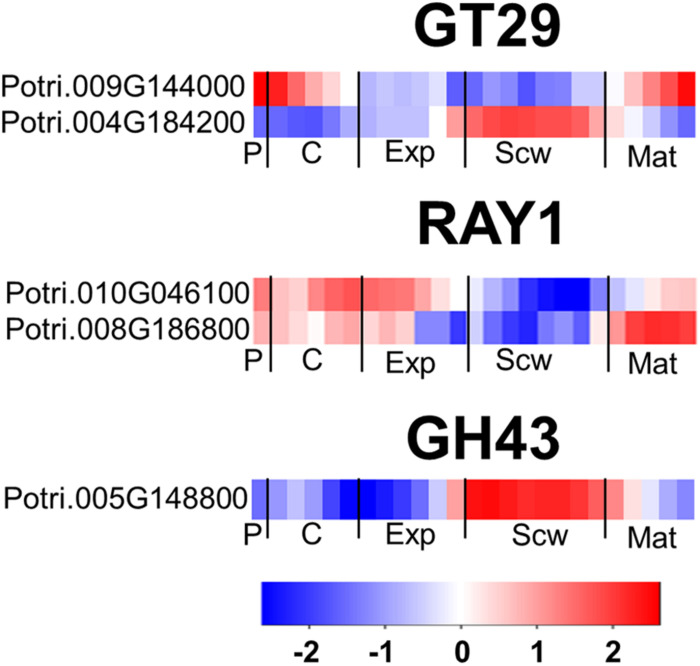
The wood expression profiles of GT29, RAY1, and GH43 *Populus* orthologs. Heatmaps depicting the relative expression of GT29A, RAY1, and GH43 proteins from *Populus tremula* in phloem (P), cambium (C), xylem expansion zone (Exp), xylem secondary cell wall formation zone (Scw), and xylem maturation zone (Mat). Expression values are scaled per gene so that expression values above the gene average are shown in red and below average in blue. *n* = 4 biological replicates.

**FIGURE 8:**
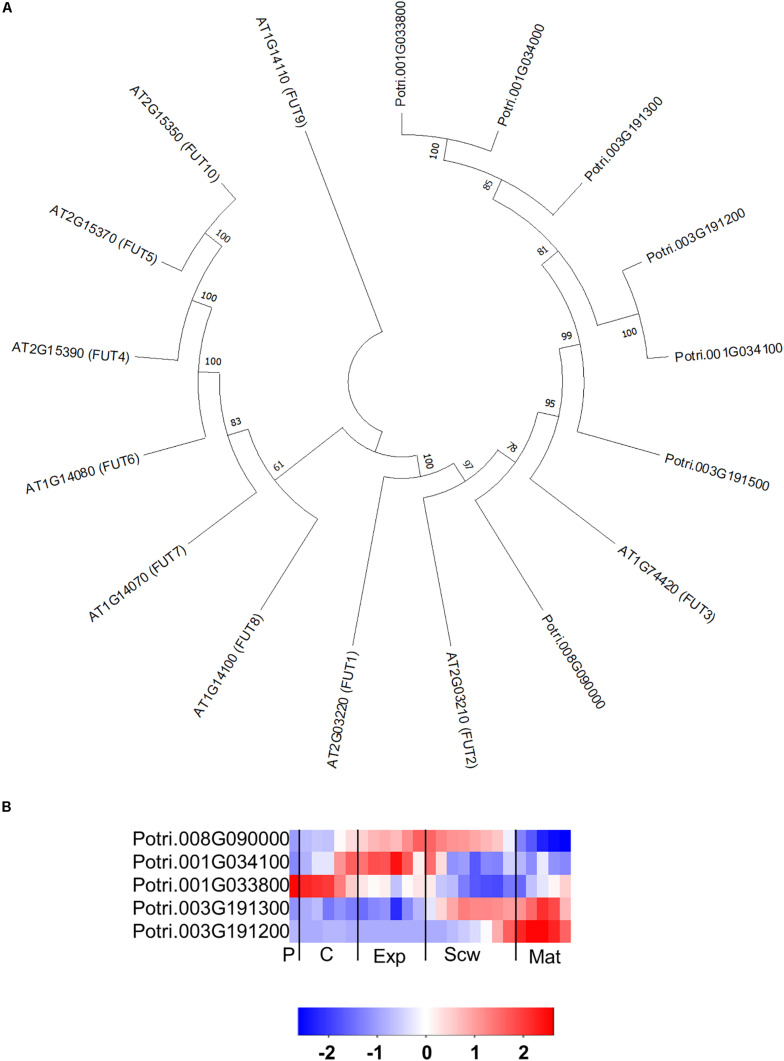
The phylogeny and expression of the GT37 (FUT) proteins in *Populus.* **(A)** Phylogenetic tree describing the phylogeny of the GT37 (FUT) proteins from *Populus trichocarpa* and *Arabidopsis thaliana*. Phylogenic trees were constructed using maximum likelihood method of MEGA-X in default mode with bootstrap test of 1000 replicates. The numbers beside the branches correspond to % bootstrap values. **(B)** Heatmap depicting the relative expression of GT37 (FUT) proteins from *Populus tremula* in the phloem (P), cambium (C), xylem expansion zone (Exp), xylem secondary cell wall formation zone (Scw), and xylem maturation zone (Mat). Expression values are scaled per gene so that expression values above the gene average are shown in red and below average in blue. *n* = 4 biological replicates.

**FIGURE 9:**
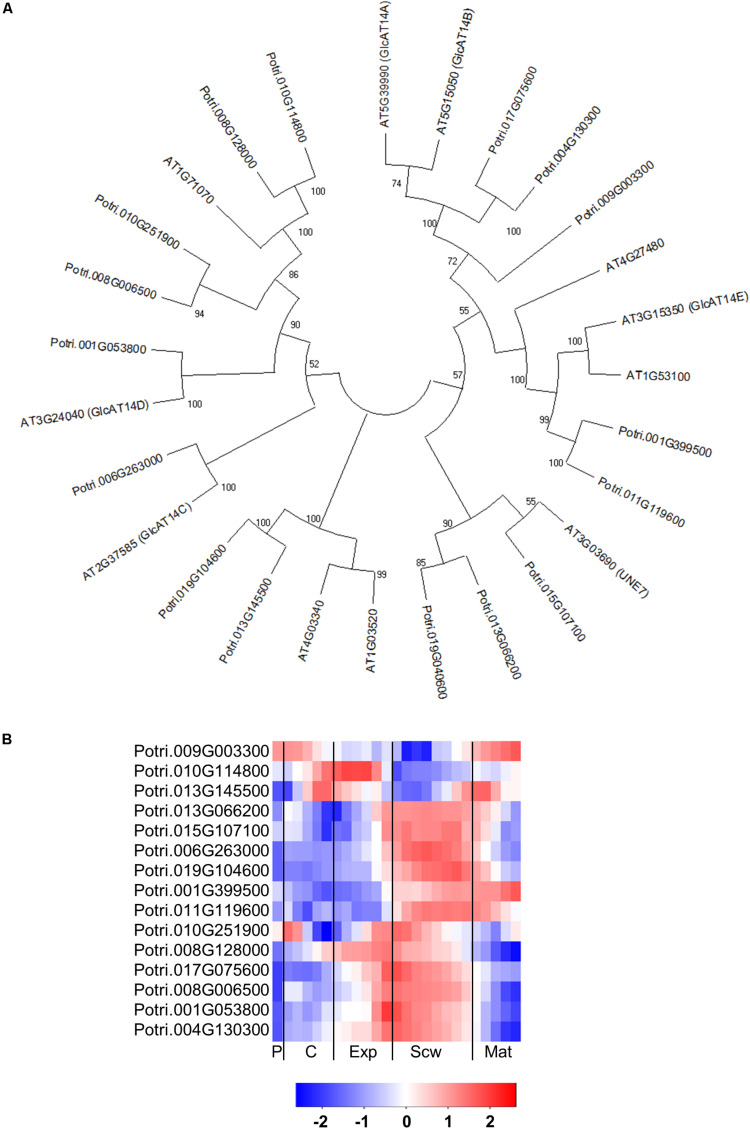
The phylogeny and expression of GT14 (GlcAT14) proteins in *Populus.* **(A)** Phylogenetic tree describing the phylogeny of GT14 (GlcAT14) proteins from *Populus trichocarpa* and *Arabidopsis thaliana*. Phylogenic trees were constructed using maximum likelihood method of MEGA-X in default mode with bootstrap test of 1000 replicates. The numbers beside the branches correspond to % bootstrap values. **(B)** Heatmap depicting the relative expression of GT14 (GlcAT14) proteins from *Populus tremula* in the phloem (P), cambium (C), xylem expansion zone (Exp), xylem secondary cell wall formation zone (Scw), and xylem maturation zone (Mat). Expression values are scaled per gene so that expression values above the gene average are shown in red and below average in blue. *n* = 4 biological replicates.

**FIGURE 10:**
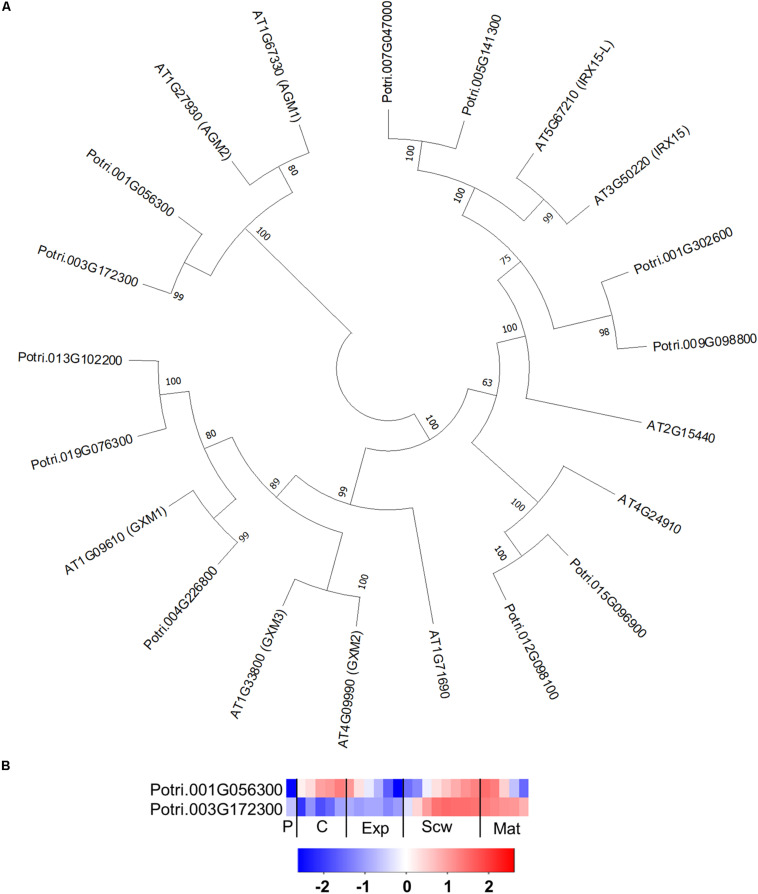
The phylogeny and expression of AGM proteins from DUF579 family in *Populus.* **(A)** Phylogenetic tree describing maximum likelihood phylogeny of the protein sequences of DUF579 family proteins from *Populus trichocarpa* and *Arabidopsis thaliana*. Phylogenic trees were constructed using maximum likelihood method of MEGA-X in default mode with bootstrap test of 1000 replicates. The numbers beside the branches correspond to % bootstrap values. **(B)** Heatmap depicting the relative expression of two AGM proteins from *Populus tremula* in the phloem (P), cambium (C), xylem expansion zone (Exp), xylem secondary cell wall formation zone (Scw), and xylem maturation zone (Mat). Expression values are scaled per gene so that expression values above the gene average are shown in red and below average in blue. *n* = 4 biological replicates.

**FIGURE 11:**
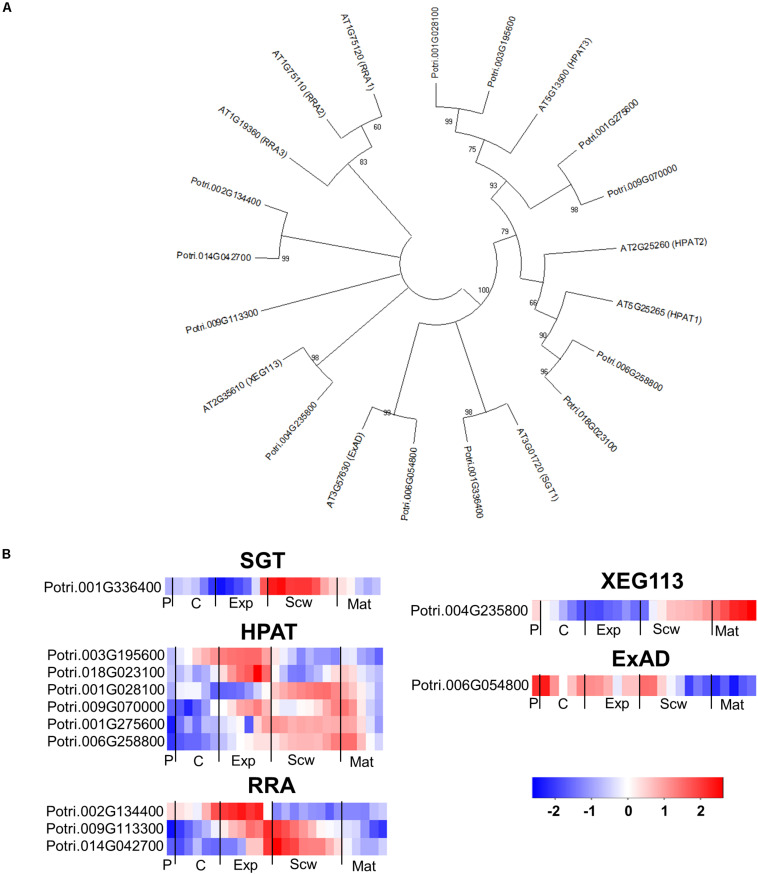
The phylogeny and expression of enzymes involved in the glycosylation of EXT proteins in *Populus.* **(A)** Phylogenetic tree describing the phylogeny of the enzymes involved in the glycosylation of EXTs from *Populus trichocarpa* and *Arabidopsis thaliana*. Phylogenic trees were constructed using maximum likelihood method of MEGA-X in default mode with bootstrap test of 1000 replicates. The numbers beside the branches correspond to % bootstrap values. **(B)** Heatmap depicting the relative expression of enzymes involved in the glycosylation of EXTs from *Populus tremula* in the phloem (P), cambium (C), xylem expansion zone (Exp), xylem secondary cell wall formation zone (Scw), and xylem maturation zone (Mat). Expression values are scaled per gene so that expression values above the gene average are shown in red and below average in blue. *n* = 4 biological replicates.

To further clarify the roles of specific HRGPs in developing wood, we compared the relative expression of the *HRGP*s to that of genes encoding enzymes predicted to be involved in HRGP glycosylation (Figures 6–11). In this analysis, the amino acid sequences of characterized glycosyl transferases from Arabidopsis known to be active in HRGP glycosylation were used to identify orthologous enzymes in *P. trichocarpa* (Sjodin et al., 2009).

The glycosyl transferase family 31 (GT31) contains enzymes catalyzing the transfer of the initial galactose moiety to the hydroxyprolines of AGPs and the subsequent elongation of the β-1,3-galactan backbone. The GT31 family from Arabidopsis has 33 members, of which 20 are likely to be involved in AGP glycosylation (Qu et al., 2008). These 20 GT31 enzymes are divided into different clades with different (predicted) activity. Clades I-II are predicted to be β-1,3 galactosyl transferases active in AGP glycosylation (Qu et al., 2008). The enzymes AtGALT31A and KNS4 from clade II have been characterized as either β-1,3- or β-1,6- galactosyl transferases (Geshi et al., 2013; Suzuki et al., 2017; Ruprecht et al., 2020). The GT31 enzymes in Clade III have been characterized as hydroxyproline galactosyl transferase enzymes that transfer the first galactose unit to hydroxyproline residues on the AGP protein backbone (Ogawa-Ohnishi and Matsubayashi, 2015). The GT31 enzymes in clade IV from Arabidopsis have not been characterized yet, but their orthologs in cotton (*Gossypium hirsutum*) were shown to be β-1,3-galactosyltransferases active in AGP glycosylation (Qin et al., 2017). Two Arabidopsis GT31 enzymes in clade V have been characterized: GALT1 is a β-1,3-galactosyl transferase for *N*-glycosylation, while GALT3 is a hydroxyproline galactosyl transferase (Strasser et al., 2007; Basu et al., 2015a). All of the members of clade VI were subsequently shown to be hydroxyproline galactosyl transferases (Basu et al., 2015a, b). The amino acid sequence of these 20 Arabidopsis GT31s involved in AGP glycosylation were used to identify *Populus* orthologs. A BLAST analysis identified 24 GT31s in the *Populus* genome including members of all six previously described clades (Figure 6A). Gene expression heatmaps showed that the members of clades I, III, and IV are expressed most strongly during secondary cell wall formation, while members of clades II, V, and VI are expressed more in the cambial and cell expansion regions, with some exceptions (Figure 6B). These results show that like the *AGP*s, the *GT31* genes are expressed in specific wood developmental zones (Figures 2, and 6B). AtGALT31A from Clade II was shown to be involved in cell division in the hypophysis of Arabidopsis embryos (Geshi et al., 2013). The closest *Populus* orthologs from clade II, except *Potri.011G151400*, are quite strongly expressed in the cambium, suggesting that they might play similar roles in cell division during secondary growth in *Populus* (Figure 6). Similarly, *Populus* orthologs from clade VI, except *Potri.16G013300*, are expressed relatively strongly in the cambium and the wood expansion zone. AtGALT2 and AtGALT5 from clade VI were previously shown to have roles in root cell expansion under salt stress, possibly involving modulation of cellulose biosynthesis (Basu et al., 2015b, 2016). These observations indicate that some *Populus* GT31s may have similar functions to their Arabidopsis orthologs at the cellular level.

The AGP side chains are synthesized by a large group of transferase enzymes in the Golgi apparatus. Two genes belonging to the GT29 family exist in Arabidopsis, 1 of which (*GALT29A*) was characterized as encoding a β-1,6 galactosyl transferase involved in AGP glycosylation (Dilokpimol et al., 2014). Five members of the GT29 family were identified in *Populus*, including two close orthologs of *AtGALT29A* (Kumar et al., 2019). One of these orthologs is expressed strongly in the phloem/cambium and maturation zone, while the other is most strongly expressed in the secondary cell wall formation zone (Figure 7). Reduced Arabinose Yariv 1 (RAY1) belongs to the GT77 family and is currently the only transferase enzyme known to transfer arabinose to AGP glycans (Gille et al., 2013). An Arabidopsis *ray1* mutant expressing a defective variant of this gene had shorter roots and smaller rosettes than wild type plants. *Populus* has two close orthologs of *RAY1* with high relative expression in the phloem, cambium, expansion zone, and maturation zone (Figure 7). In Arabidopsis, Glycoside Hydrolase family 43 (GH43) enzymes were recently shown to be Golgi-localized β-1,3-galactosidases involved in root cell expansion, possibly due to their activity toward AGP glycans (Nibbering et al., 2020). The *Populus* genome contains only one gene encoding a GH43 enzyme (Kumar et al., 2019). This gene, *PtGH43*, is strongly expressed during secondary cell wall formation unlike *AtGH43*, which is expressed most strongly during cell expansion in roots (Figure 7).

The GT37 family from Arabidopsis contains 10 members, of which FUCOSYLTRANSFERASE 4 (FUT4), FUT6 and FUT7 were shown to add fucose to AGP glycans (Tryfona et al., 2014; Ruprecht et al., 2020). The *Populus* genome contains seven FUT orthologs (Kumar et al., 2019), six of which form a distinct group in the phylogenetic tree that is separate from the Arabidopsis GT37 group (Figure 8A). This indicates that members of the GT37 family may have followed separate evolutionary expansion trajectories in *Populus* and Arabidopsis, which may be linked to the different life styles of these two species (woody perennial and herbaceous, respectively). Intriguingly, the GT37 family was the only GT family analyzed here that showed such a clear group differentiation in *Populus* and Arabidopsis. The closest Arabidopsis ortholog to the PtGT37 group is AtFUT3. AtFUT3 and the other Arabidopsis FUTs were proposed to be fucosyltransferases involved in cell wall biosynthesis because their overexpression increased fucose levels in the cell walls of transgenic Arabidopsis (Sarria et al., 2001). In support of this interpretation heterologous expressed AtFUT1, which is the closest homolog to AtFUT3, was able to transfer fucose to a galactose on the xyloglucan core (Cicéron et al., 2016). AtFUT4 and AtFUT6 have been shown to act redundantly in synthesizing fucose on AGP side chains (Tryfona et al., 2014). Recently, *in vitro* characterization of recombinant AtFUT4, AtFUT6, and AtFUT7 showed specific fucosyltransferase activity on α-1,3 galactose linked arabinofuranose (Ruprecht et al., 2020). Of the seven *PtGT37* genes, five are expressed in the developing wood zone but each one has a unique expression profile, with a peak in either the phloem/cambium, expansion zone, secondary cell wall formation zone, or maturation zone (Figure 8B).

The GT14 family exhibits β-glucuronosyltransferase (GlcAT14) activity and can thus transfer glucuronic acids to the side chains of AGP glycans. The Arabidopsis genome contains 11 GlcAT14 enzymes, of which GlcAT14A, B, C, D, and E have been characterized as β-glucuronosyltransferases (Knoch et al., 2013; Dilokpimol and Geshi, 2014; Lopez-Hernandez et al., 2020). The *Populus* genome contains 17 GT14 orthologs (Kumar et al., 2019), including close orthologs of the characterized Arabidopsis GlcAT14s (Figure 9A). Several of the *Populus* GT14 genes are expressed most strongly in the last stage of the expansion zone and/or the secondary cell wall formation zone, suggesting that they have important roles in wood biomass accumulation (Figure 9B). The glucuronic acid on AGP glycans was shown to be attached by AtGlcAT14 enzymes, and was recently shown to be important for pH-dependent calcium binding (Lamport and Varnai, 2013; Lopez-Hernandez et al., 2020). It was therefore suggested that this mechanism could play some role in intracellular calcium signaling. PtGT14 family members could play a similar role during wood development. However, it is unlikely that all of the *GT14*s expressed in developing wood are involved in HRGP glycosylation. The glucuronic acid side chains introduced during AGP glycosylation can be methylated by enzymes from the DUF579 family. Ten members of this family have been identified in the Arabidopsis genome, including some that were shown to have the glucuronoxylan methyltransferase (GXM) or arabinogalactan methylesterase (AGM) activity responsible for methylation of glucuronic acid moieties attached to AGP glycans (Temple et al., 2019). The *Populus* genome contains 11 DUF579 orthologs, including two close orthologs of the characterized AGM enzymes (Figure 10A). These two orthologs are expressed relatively strongly at the end of secondary cell wall zone, and one also has an expression peak in the cambium (Figures 10A,B). The biological function of AGP glucuronic acid methylation is unknown because an Arabidopsis *agm1agm2* double mutant exhibited no obvious defects. Temple et al. (2019) proposed that the methylation could affect pH-dependent calcium binding or prevent the addition of 4-linked sugars such as rhamnose to AGP glycan side chains, thereby potentially affecting their attachment to pectin side chains.

The glycosylation of EXTs is catalyzed by a rather small group of enzymes in Arabidopsis. The serine in the EXT SP_3_, SP_4_, or SP_5_ motifs is glycosylated by serine α-1,3-galactosyltransferase (SGT1) (Saito et al., 2014), which has one ortholog in the *Populus* genome (Figure 11A). The hydroxyprolines of the EXT motifs are glycosylated with a β-1,4-arabinose by hydroxyproline *O*-arabinosyltransferases 1-3 (HPAT1-3) (Ogawa-Ohnishi et al., 2013). The *Populus* genome contains six *HPAT* orthologs with expression peaks in all zones except the phloem and wood maturation zone (Figure 11A). The second β-1,2-arabinose unit is added by reduced residual arabinose 1–3 (RRA1-3) (Egelund et al., 2007). Both the Arabidopsis and *Populus* genomes contain three orthologs of the RRA proteins. A third β-1,2-arabinose unit is then attached by XEG113, followed by an α-1,3-arabinose attached by α-arabinosyltransferase (ExAD) (Gille et al., 2009; Moller et al., 2017). Both *XEG113* and *ExAD* have only one close ortholog in the *Populus* genome (Figure 11A). This is somewhat surprising because many Arabidopsis genes have two or more *Populus* orthologs due to the more recent genome duplication in the *Populus* lineage (Tuskan et al., 2006). All of the genes coding for enzymes predicted to be involved in EXT glycosylation have expression peaks in specific wood developmental zones, supporting the hypothesis that EXTs have specific functions during wood development (Figure 11B). Arabidopsis mutants impaired in these enzymes exhibited reduced root hair growth, establishing that the glycosylations they catalyze have important effects on tip growth in these specialized cells (Velasquez et al., 2011). It can thus be hypothesized that some EXTs and enzymes that catalyze their glycosylation may be involved in the intrusive tip growth of xylem fibers during wood formation.

### Localization of AGP- and Extensin-Linked Epitopes in Mature Wood

The gene expression analysis showed that several of the AGPs and EXTs are expressed late in wood development, indicating possible functions in mature wood. To investigate this possibility, we performed immunolabelling on hybrid aspen (*P. tremula* × *P. tremuloides*) wood cross-sections using monoclonal antibodies that bind to epitopes present in AGPs or EXTs (Supplementary Table 1).

**FIGURE 12:**
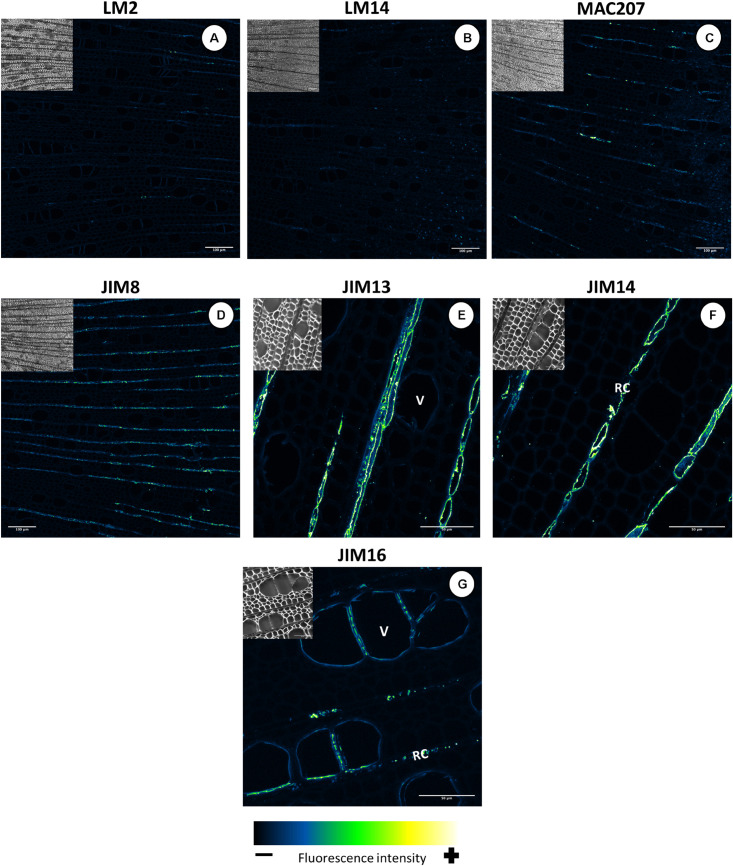
Distribution of the AGP epitopes in mature wood of hybrid aspen *Populus tremula* × *P. tremuloides*. Cross-sections of stems from 3-month-old trees were immunolabelled with a set of seven anti-AGP monoclonal antibodies: LM2 **(A)**, LM14 **(B)**, MAC207 **(C)**, JIM8 **(D)**, JIM13 **(E)**, JIM14 **(F)**, and JIM16 **(G)**. Observations were made with an inverted confocal laser scanning microscope Zeiss LSM 780 (λ_*excitation*_, 514 nm; λ_*emission*_, 535–650 nm). Fluorescence images are maximum intensity Z-projections of several focal planes. Immunolabelling were performed on sections from at least three different trees. Scale bars for LM2, LM14, MAC207 and JIM8: 200 μm. Scale bars for JIM13, JIM14, and JIM16: 50 μm. V, xylem vessel; RC, ray cells.

All of the anti-AGPs generated fluorescence signals in the cell walls of xylem ray cells (Figure 12). The signals observed from LM2, LM14, and MAC207 were appreciably weaker (Figures 12A–C) than those for JIM8, JIM13, and JIM14 (Figures 12D–F). The JIM16 signal was concentrated in clusters between adjacent xylem vessels or between vessel and ray cells (Figure 12G), possibly indicating that the JIM16 epitope has a specific function in the pit structures connecting vessels. JIM16 binds to β-1,3-galactan substituted with a single β-1,6-linked galactose residue (Ruprecht et al., 2017), which may be associated with reduced AGP side chain lengths.

**FIGURE 13:**
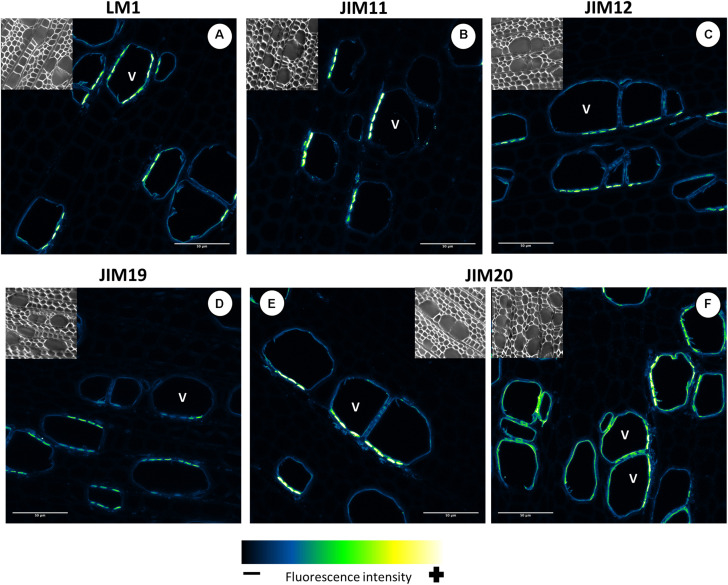
Distribution of the extensin epitopes in mature wood of hybrid aspen *Populus tremula* × *P. tremuloides*. Cross-sections of stems from 3-month-old trees were immunolabelled with a set of five anti-extensin monoclonal antibodies: LM1 **(A)**, JIM11 **(B)**, JIM12 **(C)**, JIM19 **(D)**, and JIM20 **(E,F)**. Observations were made with an inverted confocal laser scanning microscope Zeiss LSM 780 (λ_*excitation*_, 514 nm; λ_*emission*_, 535–650 nm). Fluorescence images are maximum intensity Z-projections of several focal planes. Immunolabelling were performed on sections from at least three different trees. Scale bars: 50 μm. V, xylem vessel.

All five extensin epitope antibodies displayed the same well-defined signal pattern (Figure 13). It is not clear which kinds of EXTs were labeled in this case because transcripts of classical EXTs were not detected in developing wood (Figure 3). A similar pattern was observed with all five anti-extensin antibodies, suggesting that the EXTs were correctly and fully *O*-glycosylated. The putative extensin glycosylating enzymes were expressed relatively weakly in the maturation zone (Figure 11). This may indicate that EXTs are glycosylated during active cell wall biosynthesis and are long-lived cell wall components. The similar signal patterns of the EXTs also suggest that the entire EXT glycan is easily accessible to antibodies. The EXT epitopes were observed in the cell walls of xylem vessels, forming clusters oriented toward the ray cells (Figures 13A–E) or neighboring xylem vessels (Figure 13F). These signal clusters were similar to but more defined than those observed for the anti-AGP JIM16. The location of extensin epitopes overlaps with that of the pits connecting xylem vessels and those connecting ray cells and vessels. We thus hypothesize that EXTs form part of the pit structure that enables control over solute transport between cells. These pits were shown to mainly contain cellulose, lignin, and pectins (Herbette et al., 2015); to our knowledge, the presence of EXTs or other HRGPs has not previously been reported. We speculate that EXTs play a structural role in the pits and help modulate their ultrastructure and permeability. Such a matrix modifying role would be consistent with previously proposed EXT functions in cell walls (Cannon et al., 2008; Lamport et al., 2011; Pereira et al., 2011; Chormova and Fry, 2016; Marzol et al., 2018).

### The Structure of AGP and EXT Glycans Differs Between *Populus* Stem Tissues

**FIGURE 14:**
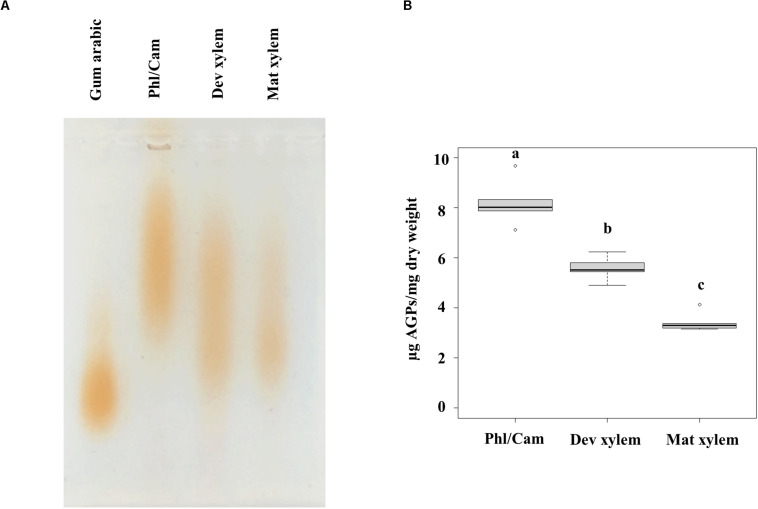
Linear AGP profiles by agarose-gel electrophoresis and quantification of AGPs by β-D-glucosyl Yariv. **(A)** Water-extracted AGPs from 15 mg dried plant material were separated by 1% (w/v) agarose gel electrophoresis followed by staining with 10 μg β-D-glucosyl Yariv reagent and further destaining in 1% NaCl. Different populations of AGPs are observed. **(B)** Colorimetric assay using β-D-glucosyl Yariv was applied to measure AGPs content as microgram per milligram of dry weight. Statistical differences were determined by one-way ANOVA, different letters (a, b and c) on each bar indicate significant differences (*P* < 0.001) according to a *t*-test. *n* = 5 biological replicates. Phl/Cam, phloem/cambium; Dev xylem, Developing xylem; Mat xylem: mature xylem.

To complement the bioinformatic and phylogenetic survey of HRGPs in the different developmental zones of *Populus* wood and the immunolocalization of HRGP epitopes in mature wood, we analyzed the structures of AGPs and EXTs in the water-soluble fractions of the phloem/cambium, developing xylem, and mature xylem. To study the AGPs present in these tissues, the water-extracted fractions from these tissues were separated by agarose gel electrophoresis and then stained with β-D-glucosyl Yariv, a reagent that binds specifically to β-1,3-galactan, which is thought to form the backbone of AGP glycans (Kitazawa et al., 2013). AGPs from developing and mature xylem migrated faster in the agarose gel than phloem/cambium AGPs (Figure 14A). Because differences in electrophoretic mobility are related to differences in the size and/or configuration of the AGPs carbohydrate moieties, these results show that the AGPs in the phloem/cambium are on average larger than those in the xylem fractions (Figure 14A). The electrophoresis-based assays also suggested that the quantity of AGPs per unit dry mass of tissue differed between the samples. To assess this quantitatively, the content of AGPs in the different stem tissues was determined using a β-D-glucosyl Yariv colorimetric assay, which confirmed that there were significant differences in AGP content between phloem/cambium, developing xylem, and mature xylem (Figure 14B). The AGP concentration was highest in phloem/cambium (8.2 μg/mg dry weight) and lowest in mature xylem (3.4 μg/mg dry weight). Overall, these results show that the AGP populations of different stem tissues differ in both size and structure, and that these differences may be related to either the composition of the glycan part and/or their interactions with other cell wall components.

**FIGURE 15:**
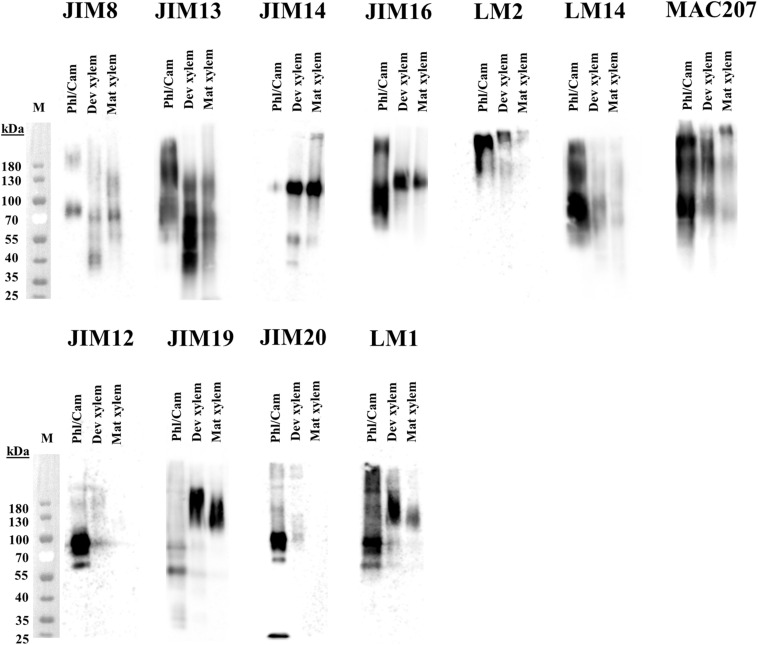
Western blot analysis of AGPs in stem tissues of aspen. Water-extracted AGPs from 2 mg dried plant material were loaded per lane, separated in SDS-PAGE according to size and transfered onto a PVDF membrane. Epitopes were detected by different anti-AGPs mAbs (JIM8, JIM13, JIM14, JIM16, LM2, LM14, and MAC207) and anti-extensins mAbs (JIM12, JIM19, JIM20, and LM1). Molecular mass (kDa) is indicated on the left. M, marker; Phl/Cam, phloem/cambium; Dev xylem, developing xylem; Mat xylem: mature xylem.

To shed further light on the differences in glycosylation structure between the tissues, the HRGPs were separated by size using SDS-PAGE and then transferred onto polyvinylidene difluoride (PVDF) membranes and probed with AGP and EXT antibodies (Supplementary Table 1). As expected given the mobility of heavily glycosylated proteins in SDS-PAGE, labeling with antibodies against both AGPs and EXTs generated broad smears rather than well-defined bands on the Western blots. The molecular masses of the corresponding glycoproteins ranged from 40 to 200 + kDa (Figure 15). There were clear differences between the studied tissues with respect to the labeling intensities of specific AGPs and EXTs, particularly between the xylem and the phloem/cambium (Figure 15). For example, the phloem/cambium extract exhibited higher signal intensities for all tested mAbs except JIM14 and JIM19, which gave stronger signals in the xylem. Interestingly, HRGP epitope heterogeneity and variation between tissues was also reported when synthetic glycoproteins containing AGPs/extensins motifs were expressed in Arabidopsis (Estevez et al., 2006). Since amino acids account for <10% of the molecular mass of AGPs, with carbohydrates comprising the remaining 90%, the differences in molecular size between the tissues are likely due to heterogeneity in the glycan structures. Alternatively, the differences could be due to the formation of complexes between AGPs and other cell wall components, which could be more extensive in phloem tissues. Associations between AGPs and pectin have been observed in several species (Oosterveld et al., 2002; Immerzeel et al., 2006; Cannesan et al., 2012; Tan et al., 2013). Interestingly, the AGPs recognized by JIM14 were barely detectable in phloem/cambium extracts. Additionally, extracts from mature xylem exhibited at most weak signals after western blotting with LM2, LM14, JIM12, and JIM20 whereas the labeling was slightly stronger in extracts from developing xylem. This suggests that these tissues differ with respect to either the carbohydrate structures of AGPs and EXTs or the accessibility of their carbohydrate epitopes to the tested mAbs. Such differences could be due to differences in cell wall organization (Eeckhout et al., 2014). The differences in LM2 labeling are particularly surprising (Figure 15 and Supplementary Table 1) because β-glucuronosyltransferase (GlcAT14) is most strongly expressed in the secondary cell wall formation zone of the wood (Figure 9B). Although transcript level does not necessarily reflect corresponding protein level, we speculate that the glucuronic acid on AGP glycans might facilitate binding to other cell wall matrix polymers (Tan et al., 2013), a possibility that is supported by the strong signal in the high molecular mass (200 kDa+) region of the western blot. Also the attachment of methyl groups to glucuronic acid by AGM (Figure 10B) may hinder the binding of LM2, and contribute to its comparatively low signal intensity in the xylem (Figure 15).

## Conclusion

A total of 157 *HRGP*s are expressed during secondary growth of *Populus* stems. Many of these genes have well-defined spatio-temporal expression patterns suggesting that they have roles in specific developmental and cell wall biosynthesis processes. The functionally important HRGP glycan structures differ between stem tissues, and these differences can be at least partly explained by the expression of different *HRGP*s and *GT*s. Additionally, the new finding that EXTs are associated with the pit regions of xylem vessels opens a new line of investigation into EXT role in xylem sap transport. The structure (porosity and thickness) of pit membranes is critical in preventing the spread of vascular pathogens and embolism making this observation relevant for understanding of stress responses in trees. The findings presented here will serve as a basis for targeted studies using RNAi and CRISPR strategies to determine the biological function of HRGPs during secondary growth of trees.

## Data Availability Statement

The raw data supporting the conclusions of this article will be made available by the authors, without undue reservation.

## Author Contributions

TA, RC, and PN planned and performed the experiments and analyzed the data. TN planned the experiments and analyzed the data. TA, RC, PN, and TN wrote the manuscript. All authors contributed to the article and approved the submitted version.

## Conflict of Interest

The authors declare that the research was conducted in the absence of any commercial or financial relationships that could be construed as a potential conflict of interest.

## Footnotes

1 https://popgenie.org

2 http://aspwood.popgenie.org/aspwood-v3.0/

3 https://www.hasselforsgarden.se

4 www.plantprobes.net

5 www.ccrc.uga.edu

6 https://fiji.sc/
