# Supplementary material for: The Spatio-Temporal Distribution of Cell Wall-Associated Glycoproteins During Wood Formation in Populus
Source: Front Plant Sci. 2020 Dec 15;11:611607. doi: 10.3389/fpls.2020.611607 (PMC7768015; doi:10.3389/fpls.2020.611607)
Supplement: Supplementary file 1 [file Data_Sheet_1.PDF]

Antibody	HRGP recognized	Epitope structure	Reference
**JIM8**	**AGP**	unknown	Pennell et al. (1991)
**JIM13**	**AGP**	(beta)GlcA1->3(alpha)GalA1->2Rha	Knox et al. (1991), Yates & Knox (1994), Yates et al. (1996)
**JIM14**	**AGP**	at least three consecutive b-1,6-linked Gal, third Gal toward the nonreducing terminus must not be arabinosylated	Knox et al. (1991), Yates & Knox (1994), Yates et al. (1996), Ruprecht et al. (2017)
**JIM16**	**AGP**	beta-1,3-linked galactan backbone when substituted with a single b-1,6- linked Gal residue	Knox et al. (1991), Yates & Knox (1994), Yates et al. (1996), Ruprecht et al. (2017)
**LM2**	**AGP**	beta glucuronic acid terminally attached to 1,6-linked galactan	Smallwood et al. (1996), Yates et al. (1996), Ruprecht et al. (2017)
**LM14**	**AGP**	unknown	Moller et al. (2008)
**MAC207**	**AGP**	(beta)GlcA1->3(alpha)GalA1->2Rha	Yates et al. (1996)
**JIM11**	**Extensin**	Probably include the third arabinose and/or following arabinose residues on the extensin glycan moiety	Smallwood et al. (1994), Yates & Knox (1994), Castilleux et al. (2020)
**JIM12**	**Extensin**	Probably comprise only the galactose on the serine residue and the two first arabinose residues on the hydroxyprolines	Smallwood et al. (1994), Castilleux et al. (2020)
**JIM19**	**Extensin**	unknown	Knox et al. (1995), Wang et al. (1995)
**JIM20**	**Extensin**	Probably include the third arabinose but not the following arabinose residues on the extensin glycan moiety	Smallwood et al. (1994), Pattathil et al. (2010), Castilleux et al. (2020), Beuder et al. (2020)
**LM1**	**Extensin**	Probably include the third arabinose and/or following arabinose residues on the extensin glycan moiety	Smallwood et al. (1995), Castilleux et al. (2020)

Supplemental table 1: List of the monoclonal antibodies used in the study

Beuder S, Dorchak A, Bhide A, Moeller SR, Petersen BL, MacAlister CA. (2020) Exocyst mutants suppress pollen tube growth and cell wall structural defects of hydroxyproline O-arabinosyltransferase mutants. Plant J. 2020;103(4):1399-1419. doi:10.1111/tpj.14808

Castilleux R, Plancot B, Gügi B, Attard A, Loutelier-Bourhis C, Lefranc B, Nguema-Ona E, Arkoun M, Yvin J-C, Driouich A. 2020. Extensin arabinosylation is involved in root response to elicitors and limits oomycete colonization. Annals of Botany 125(5): 751-763.

Knox JP, Linstead PJ, Peart J, Cooper C, Roberts K. 1991. Developmentally Regulated Epitopes of Cell-Surface Arabinogalactan Proteins and Their Relation to Root-Tissue Pattern-Formation. Plant Journal 1(3): 317-326.

Knox JP, Peart J, Neill SJ. (1995) Identification of novel cell surface epitopes using a leaf epidermal-strip assay system. Planta 196:266-270.

Moller I, Marcus SE, Haeger A, Verhertbruggen Y, Verhoef R, Schols H, Ulvskov P, Mikkelsen JD, Knox JP, Willats W. 2008. High-throughput screening of monoclonal antibodies against plant cell wall glycans by hierarchical clustering of their carbohydrate microarray binding profiles. Glycoconjugate Journal 25(1): 37-48.

Pattathil S, Avci U, Baldwin D, Swennes AG, McGill JA, Popper Z, Bootten T, Albert A, Davis RH, Chennareddy C, Dong R, O'Shea B, Rossi R, Leoff C, Freshour G, Narra R, O'Neil M, York WS, Hahn MG. (2010) A Comprehensive Toolkit of Plant Cell Wall Glycan-Directed Monoclonal Antibodies. Plant Physiol. 153:514-525.

Pennell RI, Janniche L, Kjellbom P, Scofield GN, Peart JM, Roberts K. 1991. Developmental Regulation of a Plasma-Membrane Arabinogalactan Protein Epitope in Oilseed Rape Flowers. Plant Cell 3(12): 1317-1326.

Ruprecht C, Bartetzko MP, Senf D, Dallabernadina P, Boos I, Andersen MCF, Kotake T, Knox JP, Hahn MG, Clausen MH, et al. 2017. A Synthetic Glycan Microarray Enables Epitope Mapping of Plant Cell Wall Glycan-Directed Antibodies. Plant Physiol 175(3): 1094-1104.

Smallwood M, Beven A, Donovan N, Neill SJ, Peart J, Roberts K, Knox JP. 1994. Localization of Cell-Wall Proteins in Relation to the Developmental Anatomy of the Carrot Root Apex. Plant Journal 5(2): 237-246.

Smallwood M, Yates EA, Willats WGT, Martin H, Knox JP. 1996. Immunochemical comparison of membrane-associated and secreted arabinogalactan-proteins in rice and carrot. Planta 198(3): 452-459.

Wang M, Heimovaara-Dijkstra S, van der Meulen RM, Knox JP, Neill SJ (1995) The monoclonal antibody JIM19 modulates abscisic acid action in barley aleurone protoplasts. Planta 196:271-276

Yates EA, Knox JP. 1994. Investigations into the Occurrence of Plant-Cell Surface Epitopes in Exudate Gums. Carbohydrate polymers 24(4): 281-286.

Yates EA, Valdor JF, Haslam SM, Morris HR, Dell A, Mackie W, Knox JP. 1996. Characterization of carbohydrate structural features recognized by anti-arabinogalactan-protein monoclonal antibodies. Glycobiology 6(2): 131-139.
